# Peruvian Maca (*Lepidium peruvianum*): (I) Phytochemical and Genetic Differences in Three Maca Phenotypes

**Published:** 2015-09

**Authors:** Henry O. Meissner, Alina Mscisz, Mieczyslaw Mrozikiewicz, Marek Baraniak, Sebastian Mielcarek, Bogdan Kedzia, Ewa Piatkowska, Justyna Jólkowska, Pawel Pisulewski

**Affiliations:** 1 Faculty of Health Studies, Charles Sturt University & Therapeutic Research, TTD International Pty Ltd, 39 Leopard Ave., Elanora, QLD 4221, Australia;; 2 Research Institute of Medicinal Plants, 27 Libelta St., 61-707 Poznan, Poland;; 3 Faculty of Food Technology, Cracow University of Agriculture, 122 Balicka St., 30-149 Krakow, Poland

**Keywords:** Glucosinolates, HPLC profiles, Hypocotyl colours, Peruvian Maca, Phenotypes

## Abstract

Glucosinolates were previously reported as physiologically-important constituents present in Peruvian Maca (*Lepidium peruvianum* Chacon) and linked to various therapeutic functions of differently-colored Peruvian Maca hypocotyls. In two separate Trials, three colours of Maca hypocotyls “Black”, “Red” and “Yellow” (termed “Maca phenotypes”), were selected from mixed crops of Peruvian Maca for laboratory studies as fresh and after being dried. Individual Maca phenotypes were cultivated in the highlands of the Peruvian Andes at 4,200m a.s.l. (Junin and Ninacaca). Glucosinolate levels, chromatographic HPLC profiles and DNA variability in the investigated Maca phenotypes are presented. Genotypic profiles were determined by the ISSR-PCR and RAPD techniques. Compared to the Black and Red phenotypes, the Yellow phenotype contained much lower Glucosinolate levels measured against Glucotropaeolin and m-methoxy-glucotropaeolin standards, and exhibited different RAPD and ISSR-PCR reactions. The Red Maca phenotype showed the highest concentrations of Glucosinolates as compared to the Black and Yellow Maca. It appears that the traditional system used by natives of the Peruvian Andean highlands in preparing Maca as a vegetable dish (boiling dried Maca after soaking in water), to supplement their daily meals, is as effective as laboratory methods - for extracting Glucosinolates, which are considered to be one of the key bioactive constituents responsible for therapeutic functions of Peruvian Maca phenotypes. It is reasonable to assume that the HPLC and DNA techniques combined, or separately, may assist in determining ID and “Fingerprints” identifying individual Peruvian Maca phenotypes, hence confirming the authenticity of marketable Maca products. The above assumptions warrant further laboratory testing.

## INTRODUCTION

In 1981 Johns (1) stipulated, that high concentrations of Glucosinolates (benzyl and p-methoxybenzyl glucosinolates as well as their isothiocyanate derivatives in particular) in Peruvian Maca hypocotyls (*Lepidium peruvianum* Chacon – *L. peruvianum*), represent a major functional group of biologically active compounds and has suggested a link between the presence of Glucosinolates and traditionally acknowledged fertility-enhancing properties. Gonzales (2) confirmed this observation and stipulated further, that Glucosinolates in differently-coloured hypocotyls may be linked to specific physiological and therapeutic properties of individual Maca phenotypes (3). The above assumption have been confirmed in clinical research by Gonzales and his Research Group (4), who, in a series of studies on male subjects (5-7) demonstrated an existence of differences in the effects of Peruvian Maca phenotypes, which may be linked to those levels of Glucosinolates detected in Black, Red and Yellow phenotypes (2).

Since there is no information on a laboratory procedure which could help in distinguishing individual phenotypes in mixed crops of Maca phenotypes, an attempt has been made in this paper, to determine both levels of Glucosinolates, together with chromatographic profiles in three selected Maca phenotypes, “Black”, “Red” and “Yellow”. A comparison of laboratory extraction methods with traditional Maca preparation system and their effect on the resultant yields of extracted Glucosinolates was also made.

## METHODS

### Material

**Maca** (*L. peruvianum*): The plant species published in the catalogue of the flowering plants and gymnosperms of Peru (8) was phytochemically characterised in the previous paper from this series (9). In two separate Trials, several samples of hypocotyls representing Maca phenotypes “Yellow”, “Black” and “Red” were separated from mixed harvested Maca crops grown in two locations on the highlands of the Peruvian Andes (Junin and Ninacaca).

**Trial I:** Hypocotyls representing “Black” and “Red” phenotypes of Peruvian Maca in a cultivated Maca crop located in Ninacaca at 4,200 m a.s.l. (Departamento Pasco) were sampled by a Research Group led by Dr. Gustavo F. Gonzales from Faculdad de Ciencias y Filosofia, Universidad Peruana Cayetano Heredia, Lima, Peru. Maca hypocotyls were dried to approximately 10% moisture and the sampling procedure was described in detail by Gonzales *et al*. (3). Sub-samples of the intact hypocotyls of both phenotypes were sent to the Faculty of Food Technology University of Agriculture Cracow, Poland (Laboratory A), Research Institute for Medicinal Plants, Poznan, Poland (Laboratory B), and Analytical Division, Plant Science, SCU, Lismore, Australia (Laboratory C) for Glucosinolate analysis.

**Trial II:** Hypocotyls of three Maca phenotypes labelled “Yellow” “Black” and “Red” were sampled from a mixed Maca crop, harvested from an organically-certified (10) cultivation site in Junin (Junin plateau) situated at 4,200 m a.s.l. Samples of freshly-harvested hypocotyls representing three Maca phenotypes were collected and packed on site, in air tight, double-walled plastic bags. This was then followed by approved air transport and delivery some 80hr after being harvested, to a Laboratory in Poznan, Poland for further analytical procedure.

### Analytical Procedures

**Trial I:** The glucosinolate content in hypocotyls of the two dried Maca phenotypes (*L. peruvianum*) “Red” and “Black” originating in Ninacaca were comparatively examined using HPLC methods: Laboratory A - by Michalski *et al.* (11) and Kraling *at al.* (12); Laboratory B - by Li *et al.* (13) adopted with modifications, so as to adjust it to internal laboratory conditions (14) and Laboratory C - using the procedure based on the method by Piacante (15) and Mc Lure (16) with laboratory modifications described in the previous paper from this series (9, 17).

The common glucosinolate Glucotropaeolin (Benzylglucosinolate – Laboratory A and B) and Sinigrin (2-propenyl glucosinolate derived from black mustard - Laboratory C) were used as reference standards for Glucosinolates.

The data was presented as means ± SD (n=2). One-way, parametric analysis of variance (Statistica v. 8.1, StatSoft, Inc., Tulsa, OK, USA) was applied for testing the differences between experimental treatments. The Duncan’s test was used for the identification of statistically significant differences at a level of p<0.05.

The HPLC resolutions for the two Maca phenotypes were extended into 3D Maca spheroids (Laboratory C), both assessed for possible use as “fingerprints” which assisted in distinguishing the two Maca phenotypes.

**Trial II:** Laboratory B: The glucosinolate contents, HPLC spectra and genetic variability in hypocotyls of the three Maca (*L. peruvianum*) phenotypes: Yellow, Black and Red were studied. In this Trial, all the intact fresh hypocotyls (8 - 10 of the three phenotypes each) delivered to the Laboratory in a chilled state were placed in a refrigerator at 4°C (+/- 1°C). Then, the three fresh hypocotyls from each phenotype sample were used in DNA study and the three remaining hypocotyls were kept under refrigeration for further chemical analysis as “fresh”, with the remaining hypocotyls dried at 37°C (+/- 1°C) to approx. 92% DM for use in Glucosinolate determination.

**(a) Glucosinolate analysis in fresh and dry Maca hypocotyls using HPLC procedure:** Samples of 5 g dry and 15 g fresh Maca hypocotyls of the three Maca phenotypes: Yellow, Black and Red were grated and pulverized under liquid nitrogen into fine powder using a Warring blender followed by mortar and pestle grinding. The method and the protocol described by Li *et al.* (13) with modifications (13), identical to the procedure used in Trial I for Laboratory B, was applied. Three 1 g samples from each powdered hypocotyl were extracted in methanol, followed by purification using a solid-state extraction method and Glucosinolate detection at 235nm against Glucotropaeolin as an external standard.

**(b) Traditional Maca preparation procedure and yields of extracted Glucosinolates:** Glucosinolate extraction efficiency was assessed by comparing the traditional system of dried Maca preparation (soaking and boiling in water) used by natives of the Peruvian highlands, with a solvent extraction system, used in laboratories. About 5 g dried Maca hypocotyls: Yellow, Black and Red phenotypes were reduced to powder in liquid nitrogen. Three pulverised phenotype samples (1 g each) were used for HPLC analytical procedure with two stage extraction protocol as follows:

In the 1^st^ extraction stage, Glucosinolates were extracted from 1 g pulverised samples at room temperature by shaking for 2 h with 30 ml of:

Distilled H_2_O (100%)

H_2_O: EtOH 50:50 (v/v)

H_2_O: EtOH 4:96 (v/v)

Samples from the first two extraction options (H_2_O and H_2_O: EtOH 50:50, v/v) were filtrated and then lyophilized under reduced pressure in -50°C. The third sample (H_2_O: EtOH 4:96, v/v) was filtrated and evaporated to dryness under reduced pressure in 40°C for further HPLC analysis.

In the 2^nd^ extraction stage, the filtered Maca solid fractions separated after the 1st stage. “Cold extractions” were individually dissolved in 30 ml of distilled water and boiled under reflux for 15 min, followed by filtration and evaporation under reduced pressure in -50°C.

HPLC procedure: Each individually lyophilized sample from the 1^st^ and the 2^nd^ extraction was dissolved in 5 ml of distilled water. SPE C18 Bakerbond cartridge was used for purification. The SPE column was conditioned by 2 ml of methanol, followed by 8 ml of water. 1 ml of sample was added on SPE column, and glucosinolates were eluted with water (0.5 ml of water, followed by four elutions with 2.0 ml water). All examined solutions were passed through the SPE cartridge and obtained elutes were collected and diluted to 10 ml with water. HPLC analysis was performed on Agilent 1100 HPLC system, equipped with photodiode array detector. The mobile phase was as follows: mixture aqueous phosphate buffer (pH=7 KH_2_PO_4_ 9.078g/l Na_2_HPO_4_ 11.188g/l) and MeOH (3:7 v/v) with 0,005 M tetraoctylamonium bromide. A Lichrospher RP 18 (250 mm × 4 mm; 5 μm) column (MERCK KGaA) was used for all separations (in isocratic eluting conditions). All separations were performed at a temperature of 30°C. The flow rate was adjusted to 0.75 ml/min with the detection wavelength set to DAD at λ=235 nm; 20 μL of samples were injected. All peaks were assigned by spiking the samples with standard compound and then comparing them with the UV-spectra and their retention time.

The adopted laboratory procedure was internally validated, including the assessment of selectivity, reproducibility, linearity and accuracy of the laboratory technique used. Two external standards: Glucotropaeolin (Glucotropaeolin RM from PhytoLab GmBH) and Sinigrin (as Sinigrin hydrate from Sigma-Aldrich), were both used as reference Glucosinolate markers in analysed Maca phenotypes.

**(c) Analysis of Genetic variability in Peruvian Maca hypocotyls.** Extraction of viable protein and isolated genomic DNA was performed on fresh intact Maca hypocotyls delivered to the laboratory in a chilled state (10°C to 15°C) some 80hrs from their harvest in the Andean highlands. Genotypic variability was determined by means of the ISSR-PCR (Inter-Simple Sequence Repeats polymerase chain reaction) and RAPD (Randomly Amplified Polymorphic DNA) techniques.

DNA extraction: Samples of fresh Maca hypocotyls were quickly frozen in liquid nitrogen and then homogenized in a mortar to obtain a fine powder. The ground tissue (0.3 g) was placed in 1 ml 2% CTAB buffer (cetyltrimethylammonium bromide) and then extracted with 100 μl chloroform: isopropanol. After incubation in 65°C for 30 min, all samples were centrifuged at 2400 rpm for 10 min. in 4°C. DNA was precipitated with 650 μl of cold isopropanol. After 60 min of incubation, samples were centrifuged at 12000 rpm for 10 min. in 4°C. The pellet was gently mixed with 1 ml 0.2 M sodium acetate and 75% ethanol. After mixing samples were spun at 12000 rpm in 4°C for 10 minutes, Supernatant was discarded and the pellet was air-dried for 20 minutes. The pellets then resuspended in 100 μl of deionized water. DNA at 100 ng/µl concentrations was prepared for further analysis.

RAPD/ISSR-PCR: The reaction consisted of 50 ng DNA, 20 pmol of each primer (OPL Kit, OPERON or single RAPD primer, OLIGO), and 7.5 μl of PCR Mix (Fermentas, 2×). The final volume of the reactions was 15 μl. Primer combinations were chosen randomly. An MJ PTC-100 DNA thermal cycler was used to amplify the DNA fragments. The cycler was programmed as follows: 20 cycles of 95°C for 300 sec, 92°C for 90 sec, 35°C for 90 sec and 72°C for 120 sec followed by 20 cycles of 90 sec at 92°C, 90 sec. at 38°C and 120 sec at 72°C for denaturing, primer annealing and primer extension, respectively. A final 300 sec extension at 72°C was carried out after the cycles were completed. The amplified products were separated by electrophoresis on 1.5% agarose gel in 1xTBE buffer, stained with ethidium bromide and on 6% acrylamide gel by silver staining. VL vilber Lourmat Transiluminator TFX-20M system was used for gel documentation.

**(d) Bacterial contamination of hypocotyls in tested Peruvian Maca phenotypes.** Since routine bacterial testing of different batches of Maca phenotype mixtures on arrival at the processing plant in Lima, showed inconsistency in total bacterial counts, an attempt has been made to investigate any possible differences which may exist in the susceptibility of Peruvian Maca phenotypes to bacterial contamination. The degree of microbiological contamination in the three Maca phenotypes was tested using a standard detection technique adopted in Laboratory B for herbal material and

## RESULTS

### Trial I

The two Peruvian Maca *(L. peruvianum)* phenotypes Red and Black collected in Ninacaca were analysed as dried hypocotyls. Irrespective of the laboratory where analyses were performed and the method used, in each case, distinctively higher Glucosinolates concentrations (P<0.01) were detected in Red phenotype hypocotyls as compared to the Black Maca specimens (Table 1). There were also statistically highly significant differences in Glucosinolate results reported for Red Maca phenotypes, with Laboratory B detecting lower values as those reported by Laboratory A (P<0.01), while there were no statistical differences in results reported by both laboratories for the Black Maca phenotype (P>005). Laboratory C has not provided sufficient data for statistical analysis, although the reported results were close to the values reported by Laboratory B for both phenotypes.

**Table 1:** Glucosinolates concentration (g % and ± SD) in two Peruvian Maca hypocotyls (*L. peruvianum*) phenotypes Red and Black collected in Ninacaca location at the altitude 4,200 m a.s.l. and analysed in three Laboratories using three analytical methods*

Maca Phenotype	Laboratory A^1^	Laboratory B^2^	Laboratory C^3^

Red hypocotyl	0.993 (± 0.187) a I	0.457 (± 0.027) a II	0.375
Black hypocotyl	0.085 (± 0.004) b III	0.072 (± 0.054) b III	0.088

* Values in each column with unlike lower case letters indicate an existence of differences between Red and Black Maca phenotypes at statistically highly significant level (*P*<0.01) within each Laboratory, while unlike Roman numbers within the rows indicate significant differences between results obtained by the use of different methods used for analysis of Maca phenotypes in Laboratory A and B only at *P*<0.05 level.

1 Faculty of Food Technology University of Agriculture Cracow, Poland (Adoption of the method by Michalski *et al.* (1995) and Kraling *at al*. (1990) - determined against glucotropaeolin standard);

2 Research Institute of medicinal Plants Poznan, Poland (Adoption of the method by Li *et al.* (2001) - determined against glucotropaeolin standard);

3 Analytical Division, Plant Science, SCU, Lismore, Australia (Using the method by Piacante *et al.* (2002) and Mc Lure (2004)-determined against Sinigrin standard). Statistic not available due to single determination supplied only.

**Figure 1:**
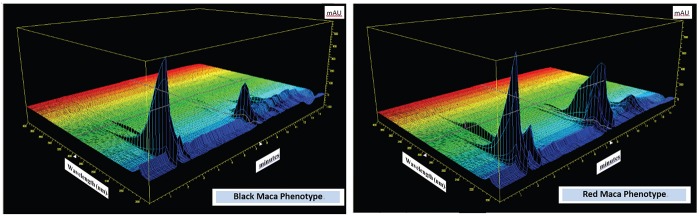
3D HPLC Resolutions: Maca *(L. peruvianum)* phenotypes “Black” and “Red” grown at altitude 4,200 m a.s.l. Chromatographic profiles of the two Peruvian Maca phenotypes generated with the use of 3D plot mode of HP Chemstation software from analysis of Glucosinolates (against Sinigrin standard) selected as an active compound characterising the two phenotypes of cultivated Peruvian Maca. [Glucosinolates determined in Black Maca = 0.088% and in Red Maca = 0.375%].

There were distinctive differences in the 3D spectra between the two tested Peruvian Maca (*L. peruvianum*) phenotypes Black and Red, generated with the use of the 3D plot mode of HP Chemstation software as presented in Figure 1. The most pronounced difference is noted in the area of peaks representing Glucosinolates (9 to 10 min on the chart). There are many unidentified peaks on the chart, for which standards were not available in the Laboratory at the time when this work was conducted.

### Trial II

**(a) Glucosinolates in fresh and dry Maca hypocotyls.** There were statistically significant differences (*P*<0.01) between Glucosinolate levels determined in three Peruvian Maca *(L. peruvianum)* phenotypes Red, Black and Yellow, collected in Junin, with fresh hypocotyls showing significantly higher values, as compared to those analysed after the hypocotyls were dried (Table 2). Distinctively higher Glucosinolate concentrations were detected in dry Red phenotype hypocotyls as compared to the dried Black and Yellow Maca specimens (*P*<0.01), while fresh Red and Black Maca hypocotyls were not significantly different in the recorded values (*P*>0.05). Both Red and Black fresh Maca specimens showed nearly ten times higher content of Glucosinolate than the fresh Yellow Maca. Those differences between the fresh Red and Black hypocotyl values and the fresh Yellow Maca samples were statistically, highly significant (*P*<0.01).

There were no statistically significant differences in Glucosinolates contents between dry both Red and Black Maca phenotypes originating in Junin and Ninacaca (*P*>0.05) when analysed by the same detection method used in Laboratory B (Table 2).

**Table 2:** Glucosinolates concentration (g %) in three phenotypes of Peruvian Maca hypocotyls (*L. peruvianum*) Red, Black and Yellow collected in Junin location at the altitude 4,200 m a.s.l. and analysed1 as fresh or after being dried*

Maca Phenotype	Fresh hypocotyls^1^ Junin	Dry hypocotyls Junin	Dry hypocotyls Ninacaca	± SD

Red	1.656 a I	0.357 a II	0.457 a II	± 0.278
Black	1.684 a I	0.085 b III	0.072 b III	± 0.196
Yellow	0.193 b II	0.153 b II	n.s.	± 0.091

a Values in the columns with unlike lower case letters within the columns indicate an existence of differences between Red, Black and Yellow Peruvian Maca phenotypes at statistically highly significant level (*P*<0.01), while unlike Roman numbers within the rows indicate significant differences (*P*<0.01) between results obtained from hypocotyls analysed as fresh or after being dried. n.a., no sample available for analysis.

1 Laboratory B - Research Institute of Medicinal Plants, Poznan, Poland (Glucosinolates results obtained with the use of the method by Li *et al.* (2001) and determined against Glucotropaeolin as an external standard).

**Figure 2:**
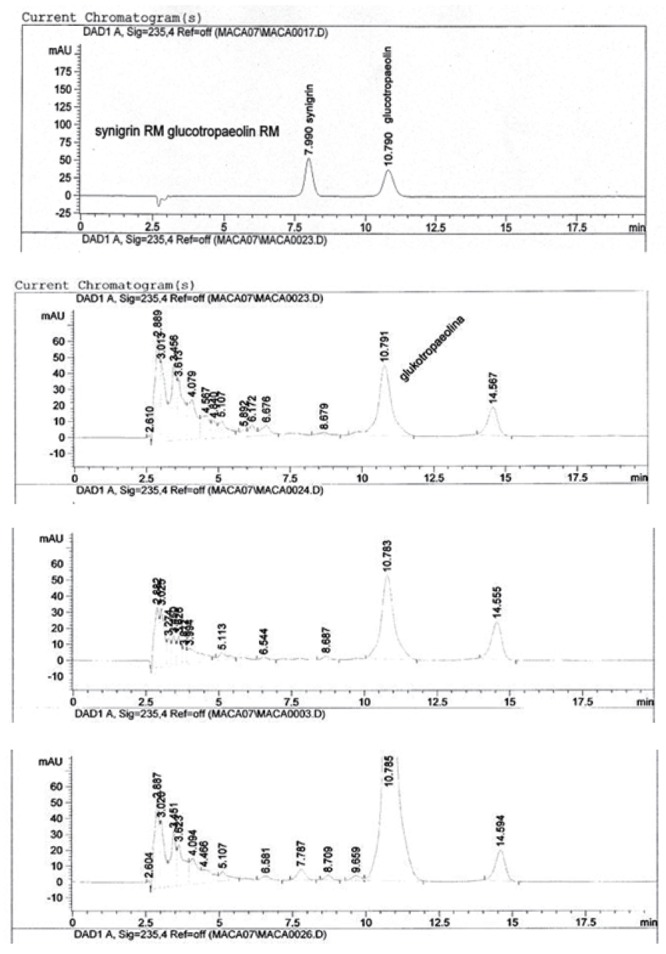
HPLC resolutions and retention times for Synigrin (7.9 min) and Glucotropaeolin (10.8 min) Standards with corresponding Glucosinolates resolution spectra for dried Peruvian Maca (*L. Peruvianum)* phenotypes “Yellow”, “Black” and “Red” showing corresponding concentrations of Glucosinolytes determined as Glucotropaeolin equivalent and one, yet unindentified peak on all phenotype charts at 14.5 min as well as two unique peaks in “Red” phenotype chart surrounding 7.7 min and 9.6 min, which after identification could be used as a unique phytochemical fingerprint for Red Peruvian Maca.

There was no Sinigrine determined in all the analysed fresh and dry Maca phenotypes originating in Junin as shown on the example of HPLC resolution spectra (Figure 2), indicating the position of Synigrin and Glucotropaeolin standards and corresponding resolutions for dried Peruvian Maca (*L. Peruvianum)* phenotypes Yellow, Black and Red. In addition to the distinctive Glucotropaeolin peak, on all the HPLC resolutions of Peruvian Maca phenotypes, there was one, yet unidentified peak, which may indicate one of the Glucosinolate derivatives positioned after Glucotropaeolin on the HPLC chart.

On the HPLC profile of Red Maca phenotype (Figure 2), there were also two unique peaks surrounding 7.7 min and 9.6 min, which need further identification.

**(b) Glucosinolates extraction efficiency using the traditional Maca preparation system.** The analytical procedure adopted in the assessment of Glucosinolate extraction efficiency from Maca phenotypes, was firstly subjected to internal validation. When Glucotropaeolin was used as a standard in the Glucosinolate detection procedure, the Relative Standard Deviation (RSD) for the determined values did not exceed 1%, and the symmetry of peak was in the range of 0.8 to 1.5. This was within the acceptable limits adopted in the EU for the type of analytical methods, as used in this study. The RSD for precision of the adopted method was 2.57%. Linearity of the relationship between peak areas and the concentration of Glucotropaeolin standards at different concentrations allowed for expressing results in the format of a regression equation (c [mg/ml] = 9.975·10^-5^·P + 8.836·10^-4^; r=0.999).

Recovery of Glucotropaeolin from the analysed Maca (*L. peruvianum)* samples ranged between 92.7% to 93.9% at RSD 0.52%, which was within the limits acceptable by EU laboratory standards. Average loss of Glucotropaeolin after a 24 hour delay in HPLC detection, was only 0.6%. This demonstrates an acceptable level of stability in those Maca preparations obtained according to the procedure adopted in this Laboratory.

Analysing results obtained from the three extraction procedures (Table 3), that extracts, after soaking in water or two other Ethanolic extraction options applied at the 1st “cold extraction” stage, contained lower levels of Glucotropaeolin, as compared to the sum of the extracted compound from the two stages of extraction (cold pre-soaking followed by boiling the remaining solids in water for 15 min.). There was no Glucotropaeolin detected in the Yellow phenotype after the 1^st^ stage of “cold extraction” (soaking for two hours) with the use of each of the three extraction mediums. Levels of Glucotropaeolin extracted in the 1^st^ stage from Black and Red phenotypes, with the use of Hydro-Ethanolic solution [H_2_O:EtOH 50:50 (v/v)], and 96% Ethanol [H_2_O:EtOH 4:96 (v/v)] were statistically not different (*P*>0.05).

**Table 3:** Total concentration of glucosinolates (g %) expressed against Glucotropaeolin standard in dry hypocotyls of three Maca phenotypes Yellow, Black and Red subjected to three different Glucosinolates extraction procedures in two systems of sample preparation (1^st^ and 2^nd^ stage)*

Extraction medium	H_2_O (100%)		H_2_O:EtOH 50:50 (v/v)		H_2_O:EtOH 4:96 (v/v)	
Maca Phenotype (Colour of hypocotyl)	1^st^ stage “cold extraction” only^1^	Sum 1^st^ + 2^nd^ stage extraction^2^	1st stage “cold” extraction” only^1^	Sum 1^st^ + 2^nd^ stage extraction^2^	1^st^ stage “cold” extraction” only^1^	sum 1^st^ + 2^nd^ stage extraction^2^

Yellow	0.00 a I	0.396 a II	0.00 a I	0.193 a II	0.00 a I	0.233 a II
Black	0.966 b III	1.430 b III	1.282 b III	1.684 b III	0.578 b II	1.835 b III
Red	0.989 b III	1.491 b III	1.197 b III	1.656 b III	0.541 b II	1.429 b III
*SD (+/-)*	*0.107*	*0.133*	*0.114*	*0.148*	*0.071*	*0.181*

* Values in each column with unlike lower case letters indicate an existence of differences between Red, Black and Yellow Peruvian Maca phenotypes at statistically highly significant level (*P*<0.01), while unlike Roman numbers within each row indicate significant differences (*P*<0.01) between results obtained from hypocotyls analysed after the 1^st^ extraction stage and the sum after the 1^st^ and the 2^nd^ extraction.

1 1^st^ stage extraction only: agitating pulverised dry Maca in one of the three extraction mediums in room temperature (“cold extraction”) for 2 hr and determining Glucotropaeolin in filtered liquid fraction. Values for Yellow Maca samples were not used for determining SD (+/-);

2 Sum of Glucotropaeolin contents detected in filtered liquid fraction after the 1^st^ stage (resembling soaking in one of the three mediums for two hours), followed by dilution of all separated individual Maca solid fractions remaining after filtration in water and boiling for 15min under reflux (2^nd^ stage).

**Figure 3:**
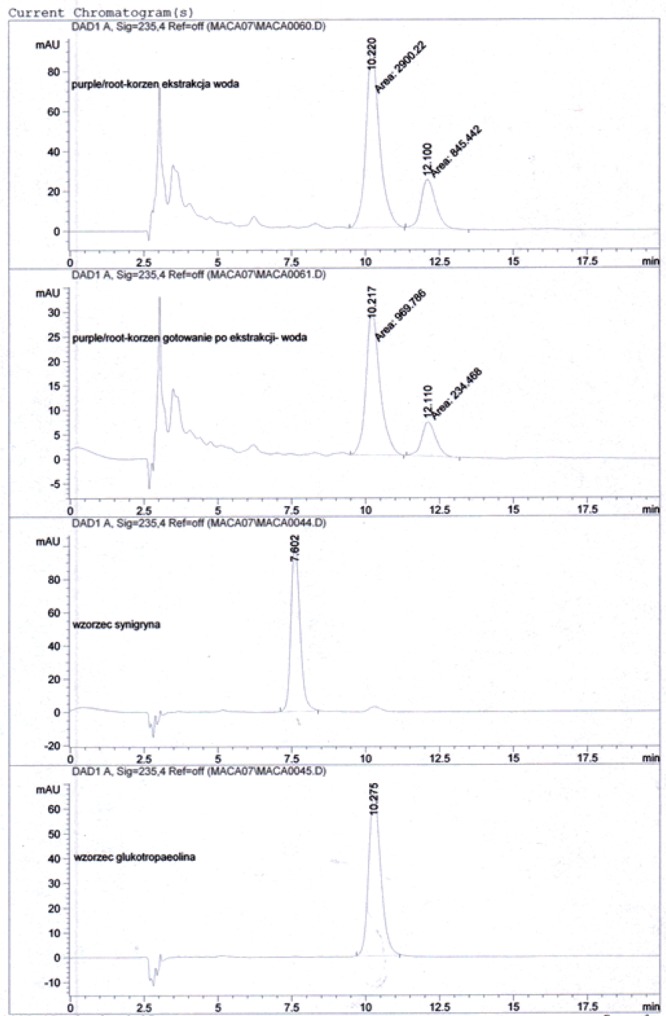
HPLC resolutions of pulverised Maca sample subjected to two stage extraction procedure: 1^st^ stage - 30 min. extraction with cold water at room temperature and then, decanted solid phase extracted again for 15 min with boiling water (2ndstage) against Glucotropaeolin and Sinigrin standards. Inserts provide chemical structure of Glucosinolates (Sinigrine & Glucotropaelin) – Side group R varies – depending on intermediary compound formed as secondary metabolite.

When Maca residues recovered from the 1^st^ “cold extraction” (for 2h at room temperature), were further boiled in water for 15 min under reflux and total glucosinolate content was expressed as the sum of the contents recorded after cold soaking (1^st^ stage) and boiling in water (2^nd^ stage), then, the sums of Glucosinolates expressed as Glucotropaeolin concentrations in all the analysed Maca phenotypes were substantially higher (*P*<0.05 and *P*<0.001) than the corresponding values recorded after the 1^st^ “cold extraction” stage (Table 3). HPLC resolutions from the two stage water extraction procedure, using the Black Maca phenotype as an example, presented on charts in Figure 3, demonstrate the absence of Sinigrin after the 1^st^ and 2^nd^ stage of extraction.

**Figure 4:**
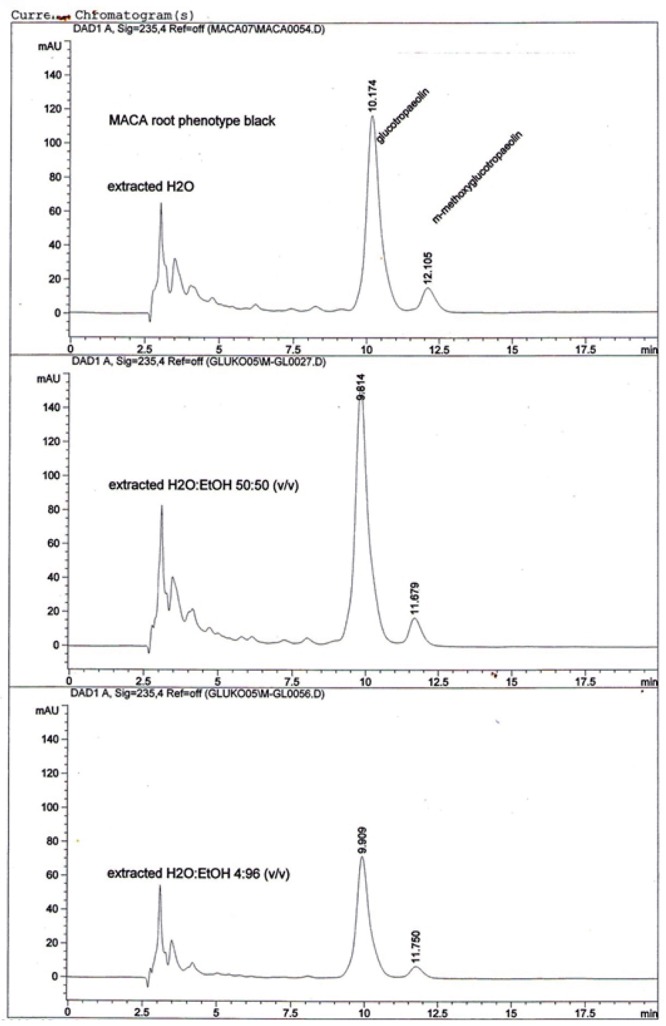
An example of HPLC resolutions for Glucotropaeolin and m-methoxyglucotropaeolin identified in Peruvian Maca (*L. peruvianum)* phenotypes “Black” after 1st-stage extraction with the use of water, ethanolic extract (50:50) and 96% Ethanol.

There was a distinctive peak appearing after Glucotropaeon HPLC traces, which was further identified to be m-methoxyglucotropaeolin, as shown on Black Maca phenotype example (Figure 4). The peak was present, although in different size, on HPLC charts from all the three extractions with the use of water, ethanolic extract (50:50) and 96% Ethanol.

In Yellow Maca phenotype, only m-methoxy-glucotropaeolin was contributing to the total Glucosinolate content. In the Black and Red Maca, quantities of detected m-methoxy-glucotropaeolin were lower than the level of detected Glucotropaeolin, which resulted in a different pattern of ratio between the two compounds in Yellow, Black and Red phenotypes as demonstrated in the last column in Table 4.

**Table 4:** Glucotropaeolin and m-methoxy glucotropaeolin concentrations (g%) determined in dried hypocotyls of Peruvian Maca (*L. peruvianum*) of three phenotypes – Yellow, Black and Red^1^ applying three extraction procedures for detection for total Glucosinolates contents*

Maca Phenotype (Colour of Hypocotyl)	glucotropaeolin	m-methoxy-glucotropaeolin	Total Glucosinolates	Contents of m-methoxy-glucotropaeolin as a percentage of the sum of Maca Glucosinolates
	[g/100g]	[g/100g]	[g/100g]	%

**Extraction: 100% H_2_O**				
Yellow	0.00^2^	0.328 c	0.328 b	100
Black	0.966 ab	0.111 ab	1.077 c	10.3 ab
Red	0.989 ab	0.150 ab	1.139 c	13.1 b
**Extraction: H_2_O:EtOH 50:50 (v/v)**				
Yellow	0.00^2^	0.141 ab	0.141 ab	100
Black	1.282 bc	0.132 ab	1.413 c	9.34 ab
Red	1.197 bc	0.189 ab	1.386 c	13.6 b
**Extraction: H_2_O:EtOH 4:96 (v/v)**				
Yellow	0.00^2^	0.038 a	0.038 a	100
Black	0.578 a	0.047 a	0.625 b	7.52 a
Red	0.541 a	0.049 a	0.590 b	8.30 a
*SD (+/-)*	*0.064*	*0.052*	*0.107*	*2.03*

* Values in each column with unlike lower case letters indicate an existence of differences between Red, Black and Yellow Peruvian Maca phenotypes at statistically significant level (*P*<0.05). Values 0.00 and 100% were not used in calculation of statistical differences.

1 Laboratory B - Research Institute of Medicinal Plants, Poznan, Poland (Glucosinolates results determined against Glucotropaeolin and m-methoxyglucotropaeolin as external standards.);

2 not detected (below detection level).

**(c) Assessment of genetic variability of the three Maca Phenotypes.** Eighteen commercial OPL primers were used in the RAPD reactions. In the RAPD exper iments with OPL primers, 4 from 18 (22%) showed genetic differences between the three different phenotypes identified by the colours of Maca hypocotyls (Black, Red and Yellow) and were genotype specific. Totally, 48 loci were amplified; monomorphic, polymorphic and genotype-specific RAPD loci were amplified in a reaction with four primers. Among 48 loci amplified, 16 ISSR loci were monomorphic (33%), 18 were polymorphic (37%) and 14 loci were genotype-specific (29%).

Eleven microsatellite primers were used in the ISSR-PCR reactions. In the ISSR-PCR reactions, 3 from 11 (27%) primers showed genetic differences between the three different colours of Maca. Totally, 49 loci were amplified; monomorphic, polymorphic and genotype-specific ISSR loci were amplified in a reaction with three primers. Among 49 loci amplified, 13 ISSR loci were monomorphic (26%), 11 were polymorphic (22%) and 25 loci were determined to be genotype-specific (51%).

**Figure 5:**
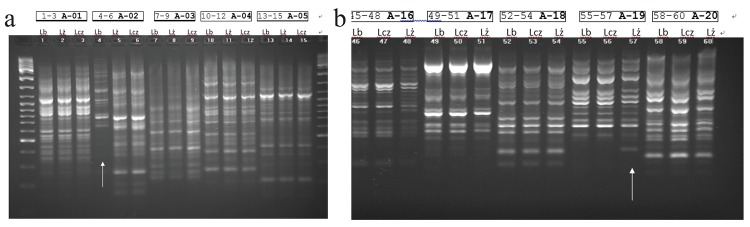
DNA extracted from three fresh Maca hypocotyls (*L. peruvianum* Chacon) Lb = Black; Lcz = Red; Lz = Yellow. Lines observed in RAPD analysis, particularly in wells 4 (Figure 5a: primer A-02  5'-TGCCGAGCTG-3') and well 57 (4th from the right - Figure 5b: primer A-19 5'-CAAACGTCGG-3') pointing with arrows at phenotypes Black (Lb) and Yellow (Lz) respectively may indicate an existence of genetic polymorphism. *Boxes above each of the three phenotypes sequence (Lb, Lcz and Lz) indicate well numbers (from – to) and corresponding primer code used for the sequence. The reaction used: 50 ng DNA, 20 pmol of each primer (OPL Kit OPERON or single RAPD primer OLIGO), and 7.5 μl of PCR Mix (Fermentas 2×). The final volume of the reactions was 15 μl.

From all hypocotyls tested in RAPD and ISSR-PCR reactions, the yellow phenotype turned out different from the others (Figure 5).

**(d) Contamination of hypocotyls in tested Peruvian Maca phenotypes.** While there were no detectable contamination of the Black phenotype Maca samples (<10/g sample), the Red Maca hypocotyls harvested from the same harvesting site show a presence of both Gam-positive aerobic *bacillus* strains and Gram-positive *cocci* strains (200/g). Of the all three phenotypes, hypocotyls of the Yellow Maca showed highest level of contamination with Gram-positive aerobic *bacillus* strains only (21,600/g).

## DISCUSSION

It was reported by Johns (1), and in an earlier study by Meissner *et al*. (9), that Glucosinolates in Peruvian Maca hypocotyls represent mostly benzyl glucosinolates and derivatives thereof. Analytical results obtained in Laboratory A, showed the presence of glucotropaeolin only (Table 1), while there were no measurable quantities of Glukonapin, Glukobrassicanapin, Progoitrynan, Poleiferyn, Sinalbin, Glukobrassycyn, and 4-Glukobrasyscyn detected in the analysed Peruvian Maca samples. Contrary to the above results, Li *et al*. (13) reported the presence of several abovementioned Glucosinolates in Maca which were not determined by the method adopted for this study in Laboratory A. However, in addition to the only Glucotropaeolin peak determined on HPLC chromatograms in Laboratory A, in Laboratory B there were two smaller un-identified peaks present on the HPLC scan, which could possibly represent longer chain aliphatic glucosinolates, or with Sulphur in the chain structure. This aspect needs to be investigated further with the use of new specific reference standards.

While there was agreement in the results reported for the Ninacaca-grown Black phenotype across the three Laboratories with the use of three different analytical methods in determining Glucosinolates contents, then significantly higher Glucosinolates values for the Red phenotype were reported from Laboratory A as compared to Laboratory B (and also substantially higher than in Laboratory C). This may indicate that with the use of the method in Laboratory A (11, 12), other unresolved Glucosinolate derivatives were detected, which possibly may be present in the Red phenotype and detected in the same position as Glucotropaeolin on the HPLC scan.

However, irrespective the Laboratory where dry Maca phenotype samples from Ninacaca were analysed and the analytical method used, the level of Glucosinolates in Red Maca phenotype was statistically significantly-higher (*P*<0.01) than in Black Maca phenotype. This trend has been confirmed by results obtained in the Trial II in analyses on dried Maca phenotypes collected in Junin, geographically-distant from the Ninacaca location. This may be an indication, that irrespective the geographic location where Maca was cultivated (both at 4,200 m a.s.l.) there may exist a similar ratio in Glucosinolates concentration between Red and Black Maca ranging from 4.2 (in Junin) to 6.3 (in Ninacaca). Such a ratio (once confirmed as a valid point for consideration) could be of possible use in designing a phytochemical model intended to identify different phenotypes of Maca used in marketable Maca products based on Glucosinolates (and/or derivatives) contents in the product.

Glucosinolate contents in fresh Red and Black Maca hypocotyls were not significantly different, and showed nearly ten times higher Glucosinolate levels than in the fresh Yellow Maca phenotype. The loss of Glucosinolates during drying was most distinctive in the Black Maca phenotype where a 20-fold reduction in concentration was recorded, while only a 4.5-fold corresponding reduction was detected in the Red phenotype. The observation on a substantially higher Glucosinolate content in fresh Maca has been also reported by Li *et al.* (13), who observed nearly six times lower content of total Glucosinolates in dry Maca hypocotyls as compared to fresh ones.

A distinctive peak appearing in HPLC resolution charts from both the 1^st^ and the 2^nd^ extraction stage was identified as m-methoxyglucotropaeolin. This was also reported by Lini *et al.* (13), who has analysed various Maca samples and products – however, without any reference to the phenotype(s) used in his study. As demonstrated in Table 4, Glucotropaeolin and m-methoxy-glucotropaeolin showed distinctive differences in the percentage value patterns recorded for the three Maca phenotypes.

Comparison of the three Glucosinolate extraction procedures, confirmed a high efficiency of Glucosinolate extraction with the use of the traditional Maca preparation procedure with water. The traditional system was as effective as a hydro-ethanolic extraction system used in the laboratory analytical procedure for Glucosinolate determination. Boiling dried and previously pre-soaked Maca hypocotyls in water, in addition to maximising the extraction of therapeutically active compounds like Glucosinolates, allows for simultaneous gelatinisation of starch granules present in Maca hypocotyls. It is generally accepted that boiling improves its acceptance as a starchy staple food component in the daily meal routinely consumed by natives of Peruvian highlands. It is broadly assumed, that boiling Maca hypocotyls and gelatinisation of its starchy component, may improve absorption and the bioavailability of poorly digestible raw starch and possibly other bio-active dietary ingredients, which are present in Maca hypocotyls.

The highest level of Glucosinolate was extracted with Hydro-Ethanolic solution, while 96% Ethanol was not satisfactory as an extraction medium. This has been supported by average recovery of Glucotropaeolin from the three types of extractions: 89.0% for water, 91.8 % for 50% hydro-ethanolic solution and only 68.1% for 96% Ethanol.

It is the authors strong belief, that until convincing proof is found as to the biochemically-identi­fied individual key active component(s) selectively extracted from known phenotype(s) of Maca, or group(s) of well-defined compounds with well-defined functionality being clinically confirmed as responsible for definite health benefits, then, the entire Maca hypocotyls with distinctive phenotypic characteristics and cohesive complexity in its compositionally unaltered form, should be used in practice as dietary Maca supplements for expected health benefits. Whole Peruvian Maca hypocotyls would then conform to their historically acknowledged and traditionally-unquestioned therapeutic properties. Several phenotype-specific traditional therapeutic effects of Peruvian Maca have been confirmed by present-day research as inducing positive gender and age related physiological responses (3, 4, 9).

It has been reported by Gonzales *et al.* (4) in clinical study on men, that Black Maca showed the best results on spermatogenesis (both semen quality and volume), memory and fatigue (energising effect), while Red Maca is distinctive in reversing the benign prostatic hyperplasia (by reducing size and negative symptoms related to enlarged prostate). It has also reversed experimentally induced osteoporosis, reduced the glucose levels and was related to the lowering blood pressure and improved health score (4).

In a series of clinical studies conducted by Meissner *et al.* on perimenopausal (19) and postmenopausal women (20, 21) with the use of standardised organic Maca blend cultivated in Junin, in which the Yellow Maca phenotype was the dominant colour (some 50% of the total), showed the predominant Yellow Maca doses significantly stimulated production of E2, suppressed blood FSH, Thyroid (T3), Adrenocorticotropic Hormones, Cortisol, and BMI. At the same time, there was a simultaneous increase in low density lipoproteins, blood Iron and an alleviation of menopausal symptoms (hot flushes and night sweating in particular), as well as noticeably increased bone density markers. Extending the use of Maca to eight months in postmenopausal women, stimulated production of both ovarian hormones, E2 and PG and resulted in a substantial reduction of menopausal discomfort felt by women participating in the study (22).

Distinctively different Glucosinolate levels in three tested Peruvian Maca phenotypes indicate, that other – yet unidentified in this study, Glucosinolates, or their derivatives, and/or other biochemical constituents other than Glucosinolates, may be involved in the expression of physiological effects of individual Maca phenotypes as observed in the earlier clinical studies on men (4) and women (21).

Based on significant differences in Glucosinolates results determined by HPLC procedure in the Red and Black hypocotyl samples grown in Ninacaca and Junin, there is a need to conduct more genetically oriented work to find out whether the chemical differences observed in differently-coloured hypocotyls of Maca phenotypes may be ascribed to genetic differences or the observed differences should be regarded as the result of natural adaptation of the plant to specific environmental conditions persisting at the 4,200m a.s.l., thus, allowing the plant’s bio-chemical matrix (in this case Glucosinolates contents in Red Maca in particular), to survive and maintain their physiological functionality and re-productive ability under harsh environmental conditions.

In the Trial I, an attempt to determine genetic differences between the Red and Black Maca phenotype originating in Ninacaca was not successful due to the problem in extracting reliable DNA from the dry Red and Black Maca hypocotyl samples, the problem described in one of the earlier papers from this series (9). The same applied to comparison of genetic profiles between the Maca phenotypes growing in two geographically-distant cultivation areas Ninacaca and Junin, which is reasonable to suppose, could induce mutations of genes susceptible to the process of adaptation to different environmental conditions where Maca is grown. This may involve, amongst other multiple factors, such changes as converting from a diploid to an amphiploid species characteristic. This aspect would need further genetic study on fresh Maca hypocotyls used in extracting quality of DNA for laboratory tests to be reliable.

The results presented in this paper show that there are genotypic differences amongst the three tested Peruvian Maca phenotypes (*L. peruvianum)* originating from the same cultivation site (Junin). A distinctive genetic variability was observed in RAPD and ISSR-PCR reactions, where the yellow phenotype was confirmed to be genetically different from the other two phenotypes. Assessment of DNA sequences made in fresh Maca hypocotyl samples, show that, with further standardisation of the methodology, it can serve as a potential tool in comparing genetic variability in products, based on different Peruvian Maca phenotypes. One would expect such an effect to exist in these distinguished by colour Maca phenotypes.

The aspect of chemical and DNA identification could be of importance in distinguishing Maca phenotypes in pulverised form, when the colours of resultant powdered hypocotyls can no longer be distinguished. It may ensure authenticity, and prevent the mislabelling of the marketable product distributed under specific Maca Phenotype names for its distinctive therapeutic and medicinal use. Examples of metabolic consequences of such unintentional or otherwise mislabelling product may be “Black Maca phenotype - for Men” and “Red Maca phenotype for Men after 50”, with the first one inducing energising properties while the second one provides a reduction of prostate hyperplasia symptoms (4).

After hypocotyls are pulverised, the currently-quoted quality as on the label of marketable Maca products (sold in the form of Maca powder or powder in capsules or in tablets), is based on the manufacturers’ assurance as to the raw material origin, its colour, proportions in a blend of phenotypes used and/or purity of phenotypes claimed to be used in the products offered to the public. After testing, the information on the label is often found to be in error – the most common being dilution of Maca with some inert powdered product (i.e. soy meal, powder from other cruciferous plants, etc.). Hence, adopting a simple routine analytical procedure to detect adulterations of Maca preparations would ensure that the purchased product will deliver expected health benefits, as per name and claims on the label of marketed Maca product. Quality assurance procedures for marketed Peruvian Maca products would need to be established in conjunction with the appropriate Regulatory Body.

The results presented in this paper provide an indication that it may be possible to establish a phytochemical and possibly a genetic fingerprint for individual Peruvian Maca phenotypes. The reason for such an assumption is demonstrated on the HPLC profile for the Red Maca phenotype, where two, yet unidentified unique peaks appearing at 7.7 min and 9.6 min, may form the basis to establish phytochemical fingerprint for the Red Maca phenotype, thus allowing to distinguish it from the other two, where these two peaks do not exist.

Also, based on results presented in this study which showed an existence of genetic differences between individual Peruvian Maca phenotypes (*L. peruvianum*), more research needs to be conducted on genetic fingerprinting of Maca phenotypes with the use of more precise laboratory techniques such as the Internal Transcribed Spacer Region of 18S-25S rDNA applied by Yang *et al*. (23, 24) in molecular phylogenetic studies of Brassica and allied genera.

## CONCLUSIONS

The results demonstrate an existence of differences in both concentrations and HPLC Glucosinolate profiles as well as in DNA sequences of the three researched Peruvian Maca phenotypes (*L. peruvianum*).

It is reasonable to suppose that genetic and phytochemical variability existing in Peruvian Maca phenotypes identified by colour of hypocotyls may be linked to different physiological responses of men and women ingesting specific Maca phenotype or their blends as observed in several clinical studies. Further research is needed to address specifically an association between phytochemical profiles of individual Peruvian Maca phenotypes, and the resultant gender-related physiological responses.

Phytochemical profiles and DNA sequences characterising differently-coloured hypocotyls representing different phenotypes in Peruvian Maca, may provide a tool in the identifying marketable Maca products, thus preventing mislabelling and/or false purity claims.

## ABBREVIATIONS

a.s.l.: Above sea level
C18: Chain length of 18 Carbons
DNA: Deoxyribonucleic acid
EthOH: Ethanol
EU: European Union
g: Gam
HPLC: High Performance Liquid Chromatograph
H_2_O: Distilled water
ID: Identity; Identifier of a unique object
ISSR-PCR: Identity; Identifier of a unique object
KH_2_PO_4_: Potassium Dihydrogen Phosphate
*L. peruvianum*: *Lepidium peruvianum* Chacon
M: Mole
MeOH: Methanol
Mix: Mixture
ml: Milliliters
Na_2_HPO_4_: Sodium hydrogen phosphate
pmol: Picomol
PMP: Peruvian Maca Project
*P*<0.01: Statistically important difference at *P*<0.01 level
*P*<0.05: Statistically important difference at *P*<0.05 level
RAPD: Randomly Amplified Polymorphic DNA
RIMP: Research Institute of Medicinal Plants, Poznan, Poland
rpm: Rotations per minute
RSD: Relative Standard Deviation
SD: Standard Deviation
v/v: On volume/volume basis
μ: Micron
μl: Microliter
μm: Micrometer
%: Percentage
°C: Degrees of Celsius
